# Dysregulated follicular regulatory T cells and antibody responses exacerbate experimental autoimmune encephalomyelitis

**DOI:** 10.1186/s12974-021-02076-4

**Published:** 2021-01-19

**Authors:** Lin Luo, Xianzhen Hu, Michael L. Dixon, Brandon J. Pope, Jonathan D. Leavenworth, Chander Raman, William R. Meador, Jianmei W. Leavenworth

**Affiliations:** 1 School of Pharmacy, Nantong University, Nantong, 226001 Jiangsu China; 2 Department of Neurosurgery, University of Alabama at Birmingham, 1600 6th Avenue South, CHB 118A, Birmingham, AL 35233 USA; 3 NIH Medical Scientist Training Program, University of Alabama at Birmingham, Birmingham, AL 35294 USA; 4 Division of Clinical Immunology and Rheumatology, Department of Medicine, University of Alabama at Birmingham, Birmingham, AL 35294 USA; 5 Department of Dermatology, University of Alabama at Birmingham, Birmingham, AL 35294 USA; 6 Department of Neurology, University of Alabama at Birmingham, Birmingham, AL 35294 USA; 7 Department of Microbiology, University of Alabama at Birmingham, 1600 6th Avenue South, CHB 118A, Birmingham, AL 35233 USA

**Keywords:** CNS autoimmunity, Experimental autoimmune encephalomyelitis, Multiple sclerosis, Humoral antibody response, Follicular regulatory T cells, Treg lineage stability

## Abstract

**Background:**

Follicular regulatory T (T_FR_) cells are essential for the regulation of germinal center (GC) response and humoral self-tolerance. Dysregulated follicular helper T (T_FH_) cell-GC-antibody (Ab) response secondary to dysfunctional T_FR_ cells is the root of an array of autoimmune disorders. The contribution of T_FR_ cells to the pathogenesis of multiple sclerosis (MS) and murine experimental autoimmune encephalomyelitis (EAE) remains largely unclear.

**Methods:**

To determine the impact of dysregulated regulatory T cells (Tregs), T_FR_ cells, and Ab responses on EAE, we compared the MOG-induced EAE in mice with a FoxP3-specific ablation of the transcription factor Blimp1 to control mice. In vitro co-culture assays were used to understand how Tregs and Ab regulate the activity of microglia and central nervous system (CNS)-infiltrating myeloid cells.

**Results:**

Mice with a FoxP3-specific deletion of Blimp1 developed severe EAE and failed to recover compared to control mice, reflecting conversion of Tregs into interleukin (IL)-17A/granulocyte-macrophage colony-stimulating factor (GM-CSF)-producing effector T cells associated with increased T_FH_-Ab responses, more IgE deposition in the CNS, and inability to regulate CNS CD11b^+^ myeloid cells. Notably, serum IgE titers were positively correlated with EAE scores, and culture of CNS CD11b^+^ cells with sera from these EAE mice enhanced their activation, while transfer of Blimp1-deficient T_FR_ cells promoted Ab production, activation of CNS CD11b^+^ cells, and EAE.

**Conclusions:**

Blimp1 is essential for the maintenance of T_FR_ cells and Ab responses in EAE. Dysregulated T_FR_ cells and Ab responses promote CNS autoimmunity.

**Supplementary Information:**

The online version contains supplementary material available at 10.1186/s12974-021-02076-4.

## Background

The generation of high-affinity Ab and avoidance of autoimmune responses after microbial infection or vaccination require precise control of the GC-Ab responses that depend on interactions between activated T_FH_ cells and GC B cells [1, 2]. Dysregulated T_FH_-GC-Ab response is the root of an array of autoimmune disorders [2]. MS is a debilitating autoimmune inflammatory disease that affects the CNS, which causes demyelination of neurons, axonal damage, and neurodegeneration. EAE induced by myelin oligodendrocyte glycoprotein (MOG) is the most commonly used animal model of MS [3]. Although emerging data have pointed out the essential contribution of T_FH_-B-GC response to EAE and MS [4–6], and myelin antigen-specific Ab response is strongly associated with disease progression in some MS patients [7], the precise role of T_FH_-B cell-Ab response in the regulation of EAE and MS remains obscure.

T_FR_ cells regulate cellular response and are also crucial for the regulation of humoral immune tolerance [8, 9]. Recent studies have emphasized this critical aspect of T_FR_ cells, as selective deletion of T_FR_ cells has a profound impact on immune responses, leading to the aberrant expansion of T_FH_ cells and excessive Ab production [10]. Like other Treg subsets, T_FR_ cells must maintain their suppressive anergic phenotype during ongoing inflammatory responses and destabilized T_FR_ cells become ex-T_FR_ cells that acquire effector cell activity [11–13]. We have recently shown that Blimp1, a transcription factor (TF) marking effector Tregs, is essential to maintain T_FR_ lineage stability, appropriate positioning in the GC, and effective regulatory activity [11]. Blimp1-deficient T_FR_ cells, but not non-T_FR_ Tregs, induce abnormal T_FH_-GC B expansion and autoantibody production by converting into effector T cells (Teff) that produce pro-inflammatory cytokines IL-17A and IFNγ [11]. However, we do not know how these Blimp1-deficient T_FR_ cells, particularly those in the specific tissue lesions, respond to the pathological conditions, like neuroinflammation in the context of EAE. Moreover, although MS patients have significantly reduced circulating T_FR_ cells compared to healthy controls (HC), residual circulating T_FR_ cells with a T_H_17 effector phenotype and impaired suppressive activity are increased in MS patients [14], while T_H_17 cells are essential to MS pathogenesis [15, 16]. It remains unclear if Blimp1 regulates the functional stability of T_FR_ cells or T_FR_ conversion into T_H_17-like cells, which may contribute to the pathogenesis of EAE and MS.

FoxP3^+^ Tregs not only maintain immune tolerance but also perform specialized functions in tissue homeostasis and remodeling by adopting genetic programs in response to the tissue microenvironmental cues [17]. Although the CNS has been viewed as an immune-privileged organ, recent studies have demonstrated an essential role of immune cells in the regulation of CNS integrity and prevention of neuroinflammation or neurodegeneration [18]. It has been recently reported that CNS Tregs are essential for the regulation of neurological recovery after ischemic stroke [19]. Currently, we do not know if a subset of Tregs exhibit CNS characteristics and regulate disease recovery in EAE and MS.

Here we explored the encephalitogenic potential of Blimp1-deficient Tregs using the MOG-induced EAE model. We observed an exacerbated EAE with the impaired recovery in mice depleted of Blimp1 in FoxP3^+^ Tregs. Blimp1-deficient Tregs, including T_FR_ cells, were converted into IL-17A/GM-CSF-producing Teff (termed as exTregs), and contributed to abnormal T_FH_ expansion and elevated Ab production, particularly IgE, in both the periphery and CNS.

## Methods

### Mice

C57BL/6J (B6), *Prdm1*^fl/fl^, *FoxP3*^YFP-Cre^, *Rosa26*^Cre-ERT2^, *Tcrα*^−/−^ (Jackson Labs), and B6SJL (CD45.1) (Taconic Farms) mice were housed in pathogen-free conditions. *Prdm1*^fl/fl^ mice were bred onto *FoxP3*^YFP-Cre^ or *Rosa26*^Cre-ERT2^ mice to generate *Prdm1*^fl/fl^*FoxP3*^YFP-Cre^ or *Prdm1*^fl/fl^*Rosa26*^Cre-ERT2^ mice, respectively. All mice were used at the age of 5 to 9 weeks unless otherwise specified. Both sexes (males or females) were randomly included for all experiments in an unblinded fashion. Generally, 5 mice were used per group, as indicated in each experiment.

### EAE induction

The procedure for the EAE induction was described previously [20]. Briefly, mice were subcutaneously injected in the right and left flanks with a total of 200 μg of MOG_35-55_ peptide (MEVGWYRSPFSRVVHLYRNGK, Cat# PEP95UNMOD, ThermoFisher) emulsified in complete Freund’s adjuvant (CFA) (Cat# F5881, MilliporeSigma) supplemented with heat-killed *Mycobacterium tuberculosis* H37Ra, and intraperitoneally injected with 200 ng pertussis toxin on days 0 and 2. Mice were monitored daily for clinical signs and scored as follows: 0, no clinical expression of disease; 1, decreased tail tone; 2, hind limb weakness or partial paralysis; 3, complete hind limb paralysis; 4, front and hind limb paralysis; 5, moribund state. The in-between scores (i.e., 0.5, 1.5, 2.5, 3.5) were given to mice with the clinical symptoms that lie between two defined scores.

### Transfer EAE

Donor mice were immunized with 200 μg MOG_35-55_ in CFA as described above. Splenocytes were isolated from donor mice, and CD4^+^ T cells were enriched before sorting CD45.1^+^CD25^−^CD44^+^CD4^+^CD3^+^ Teff and CD45.2^+^ YFP^+^(FoxP3^+^)CD4^+^CD3^+^ Tregs separately. A mixture of donor T cells (5 × 10^5^ CD4^+^ Teff and 2.5 × 10^5^ CD4^+^ Tregs) were intravenously injected into *Tcrα*^−/−^ recipients followed by immunization with MOG_35-55_/CFA and injection of pertussis toxin, as described above. For experiments involving Cre-ERT2 strains, PD-1^+^CXCR5^+^CD4^+^CD3^+^ T cells were transferred to *Tcrα*^−/−^ mice before EAE induction. These recipients were intraperitoneally injected with 1 mg tamoxifen (Cat# T5648, MilliporeSigma) emulsified in sunflower oil (Cat# S5007, MilliporeSigma) once every 24 h for 3–4 consecutive days unless otherwise specified. Mice were monitored daily after injection.

### Cell isolation

The spleen was excised and mashed between the frosted ends of two microscope slides to get a single-cell suspension. After removing red blood cells, cell suspension was passed through a 70-μm filter membrane to eliminate debris. To isolate cells from mouse brains or spinal cords, tissues were cut into small pieces (< 3 mm) and incubated in 3 ml dissociation solution [PBS supplemented with 2% FBS, 1 mg/ml collagenase/Dispase (Cat# 11097113001, MilliporeSigma) and 0.5 mg/ml DNase I (Cat# 10104159001, MilliporeSigma)] for 1 h at 37 °C with gentle shaking. Cell suspension was washed with DMEM/2% FBS, passed through a 70-μm cell strainer, and then separated on a 30% percoll gradient by centrifuging at 1400 rpm for 30 min. Cell pellets were collected for further analysis.

### Flow cytometry and sorting

Single-cell suspension was first stained with the fixable viability dye (Cat# 423105, Biolegend) at 1:1000 in PBS solution for 10 min. After washing with flow-activated cell sorting (FACS) buffer (PBS/2% FBS), cells were then incubated with Fc block (anti-mouse CD16/32 antibody) at 1:200 for 10 min, followed by staining with indicated antibody mixtures for 30 min before washing and flow cytometry analysis. For intracellular staining, cells were fixed and permeabilized using the Fixation/Permeabilization Concentrate and Diluent kit (Cat# 50-112-9060, Fisher Scientific) according to the manufacturer’s protocol, followed by incubation with Fc block and intracellular antibodies for 30 min prior to washing and flow cytometry analysis. For intracellular cytokine detection, cells were stimulated with BD Leukocyte Activation Cocktail, with BD GolgiPlug™ (Cat# B550583, BD Biosciences) for 5 h prior to staining. All of antibodies are provided in Additional file 1, and all of steps were performed at 4 °C. Cells were acquired on a BD LSRII using FACSDiva software (BD Biosciences) and analyzed with FlowJo software (Treestar). For cell sorting, single-cell suspension isolated from mouse spleens was first enriched for CD4^+^ T cells using CD4 microbeads (Cat# 130-049-201, Miltenyi Biotec). Enriched CD4^+^ T cells or isolated CNS cells were labeled with the fixable viability dye and antibodies to the surface antigens, as described above, followed by sorting on a FACSAria II using FACSDiva software (BD Biosciences).

### Enzyme-linked immunosorbent assay (ELISA)

Total IgE and anti-MOG_35-55_ IgG antibodies in mouse sera were determined by ELISA kits (Cat# 555248, BD OptEIA^TM^ and Cat# AS-54465, ANASpec Inc.), according to the manufacturers’ protocols. Total IgG antibody in mouse sera was measured using goat anti-mouse IgG (Cat# 115-005-008, Jackson ImmunoResearch) as the coating antibody, and goat anti-mouse IgG HRP (Cat# A16084, Invitrogen) as the detection antibody. The serum titers of mouse anti-MOG_35-55_ IgE were measured according to the instructions from the anti-MOG_35-55_ IgG ELISA kit (Cat# AS-54465, ANASpec Inc.) with the replacement of IgG detection antibody with IgE detection antibody.

### Immunofluorescence staining

After mice were perfused with PBS, the spinal cords were collected and immediately frozen in the cryomold containing Optimal Cutting Temperature embedding medium (O.C.T Compound, Cat# 4585, Fisher Scientific). The frozen blocks were cut into 7-μm sections that were fixed with acetone and stained with FITC-conjugated anti-mouse CD3ε antibody (Cat#100306, Biolegend) and Alexa Fluor 594-conjugated anti-mouse B220 antibody (Cat#103254, Biolegend) or Alexa Fluor 488-conjugated anti-mouse B220 antibody (Cat#103225, Biolegend) and anti-mouse IgE antibody (Cat# 553416, BD Biosciences) that was visualized using Alexa Fluor 555 goat anti-rat IgG antibody (Cat# 405420, Biolegend). Nuclei were counterstained with DAPI. Images were captured with a Leica fluorescence microscope.

### Culture of myeloid/microglia with EAE Tregs or sera

For the Treg/CD11b^+^myeloid cell co-culture assay, 1 × 10^4^ CD45^+^CD11b^+^ cells sorted from the brains and spinal cords of day 10 MOG-immunized mice were co-cultured with 5 × 10^3^ splenic YFP^+^(FoxP3^+^)CD4^+^CD3^+^ Tregs sorted from these mice in a 96-well round-bottom plate for 40 h followed by FACS analysis of Arginase-1 (Arg-1) and inducible nitric oxide synthase (iNOS) expression in CD11b^+^ cells. IL-33 (30 ng/ml) was added into CD11b^+^ cells or the co-culture groups. Isolation of myeloid/microglia from adult mouse brains was performed according to the published methods [21]. Briefly, mouse brains were harvested after perfusion and digested for 20 min in the dissociation medium (DMEM/F12 medium supplemented with 1 mg/ml papain (Cat# ICN10092180, Fisher Scientific), 1.2 U/ml dispase II (Cat# NC1136921, Fisher Scientific) and 20 U/ml DNAse I). The cell suspension was collected, filtered through a 40-μm cell strainer, and then separated on a 30-37-70% percoll gradient followed by collecting the 37-70% interphase. 5 × 10^4^ cells per well were seeded in a 24-well plate and cultured in DMEM/F12 medium with 10% FBS overnight. EAE sera were added into the culture for 48 h, followed by FACS analysis of CD11b^+^ cells. Cells treated with anti-IgG (Cat# 115-005-008, Jackson ImmunoResearch) or anti-IgE (Cat# 553416, BD Biosciences) were included as controls.

### Statistics

Statistical analyses were performed using two-tailed, unpaired Student’s *t* test, one-way or two-way ANOVA with GraphPad Prism V8 software. Error bars indicate mean ± SEM. A *P* value of < 0.05 was considered to be statistically significant (*P < 0.05, **P < 0.01, ***P < 0.001, ****P < 0.0001). No exclusion of data points was used.

## Results

### Mice with a FoxP3-specific deletion of Blimp1 develop severe EAE associated with highly activated T and myeloid cells

**Fig. 1:**
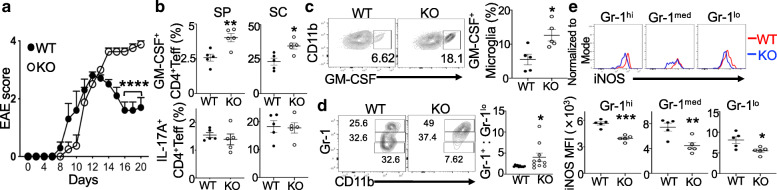
Mice with a FoxP3-specific deletion of Blimp1 develop severe EAE associated with highly activated T and myeloid cells. **a** EAE was induced with MOG_35-55_/CFA in *FoxP3*^YFP*-*Cre^ (WT) or *Prdm1*^fl/fl^*FoxP3*^YFP*-*Cre^ (KO) mice. EAE disease scores are shown. **b** Frequency of GM-CSF^+^ and IL-17A^+^ CD4^+^FoxP3^−^ Teff from EAE mice as in **a**. SP, spleen; SC, spinal cord. **c** Expression of GM-CSF in brain microglia (CD45^med^CD11b^+^) (*left*) and frequency of GM-CSF^+^ microglia (*right*). **d** CD45^hi^CD11b^hi^ subsets from the brain were gated based on Gr-1 levels. *Right*, ratios of Gr-1^+^ (Gr-1^hi^ + Gr-1^med^) to Gr-1^lo^. **e** iNOS expression in Gr-1-expressing cells from D. *Bottom*, quantitation of mean fluorescence intensity (MFI) of iNOS. Data represent one of at least three experiments (**a**–**e**: *n* = 5/group). In **d**, mice were pooled from two independent experiments. *P < 0.05, **P < 0.01, ***P < 0.001, and ****P < 0.0001 (**a**, two-way ANOVA post hoc Sidak’s multiple comparisons test; **b**–**e**, unpaired two-tailed Student’s *t* test). Bars, mean ± SEM

The expression of pro-inflammatory cytokines by dysregulated Blimp1-deficient Tregs and T_FR_ cells [11] led us to ask if Blimp1 expression in these cells may potentially regulate neuroinflammation. We then adopted the MOG_35-55_-induced EAE model [20] and mice harboring a deletion of *Prdm1* in FoxP3^+^ T cells (*Prdm1*^fl/fl^*FoxP3*^Cre^ mice) [11] compared to *FoxP3*^YFP-Cre^ (WT) mice at 5–6 weeks old. Although there were insignificant differences of disease activity at the onset of EAE for both groups of mice, there were increased peak and overall disease severity for *Prdm1*^fl/fl^*FoxP3*^YFP-Cre^ mice (Fig. 1a and Additional file 2). Of note, at around day 15 when WT mice started to recover from the disease, all of *Prdm1*^fl/fl^*FoxP3*^YFP-Cre^ mice had persistent EAE progression and succumbed to paralysis at the end of observation (Fig. 1a). Analysis of immune cells from spleens and spinal cords at the experimental endpoint revealed that FoxP3^−^CD4^+^Teff expressed more GM-CSF (but not IL-17A), and brain microglia appeared to be more activated in *Prdm1*^fl/fl^*FoxP3*^YFP*-*Cre^ mice than WT mice, as judged by an increased expression of pro-inflammatory cytokine GM-CSF (Fig. 1b, c and Additional file 3A–C for gating strategy). Among those CD45^hi^CD11b^hi^ myeloid cells that were infiltrated into the brain, more Gr1^+^ subsets than Gr1^lo^ cells were noted in *Prdm1*^fl/fl^*FoxP3*^YFP*-*Cre^ mice compared to WT mice (Fig. 1d and Additional file 3C). However, all of these myeloid cells in the brain of *Prdm1*^fl/fl^*FoxP3*^YFP*-*Cre^ mice expressed lower levels of intracellular iNOS, an enzyme with potential suppressive activity [22], than cells from WT mice (Fig. 1e). This cellular analysis suggested that mice with a specific deletion of Blimp1 in FoxP3^+^ T cells developed more severe EAE associated with highly activated Teff and CNS myeloid cells.

### Blimp1-deficient Tregs are unstable and convert into T_H_17-like cells in EAE mice

**Fig. 2:**
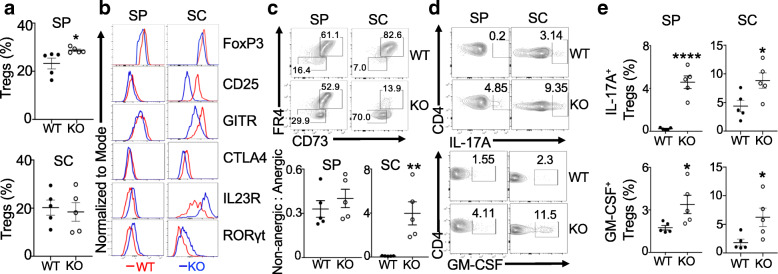
Blimp1-deficient Tregs are unstable and convert into T_H_17-like cells in EAE mice*.* **a** Frequency of splenic (SP) and spinal cord (SC) FoxP3^+^ Tregs from mice at day 20 post-EAE induction, as in Fig. 1a. **b** Expression of each molecule in SP and SC FoxP3^+^ Tregs from mice in **a**. **c** Expression of CD73 and FR4 in FoxP3^+^ Tregs (*top*) and the ratios of non-anergic (CD73^−^FR4^−^) to anergic (CD73^+^FR4^+^) population (*bottom*). **d** Production of IL-17A and GM-CSF by FoxP3^+^ Tregs. **e** Frequency of GM-CSF^+^ and IL-17A^+^ FoxP3^+^ Tregs from mice in **a**. WT, *FoxP3*^YFP-Cre^; KO, *Prdm1*^fl/fl^*FoxP3*^YFP-Cre^. Data represent one of at least three experiments (**a**, **c**, **e** *n* = 5/group). *P < 0.05, **P < 0.01, and ****P < 0.0001 (unpaired two-tailed Student’s *t* test). Bars, mean ± SEM

We next analyzed the Treg compartment from these EAE mice. Although *Prdm1*^fl/fl^*FoxP3*^YFP*-*Cre^ EAE mice had more Tregs in the spleens, the frequency of Tregs was almost similar in the spinal cords of both groups of mice (Fig. 2a and Additional file 3A, B). Compared to WT Tregs, FoxP3^+^ Tregs from the spinal cords of *Prdm1*^fl/fl^*FoxP3*^YFP*-*Cre^ EAE mice expressed reduced levels of FoxP3 and FoxP3 target molecules, including CD25, CTLA4, and GITR, but increased expression of the T_H_17 signature molecules, including IL23R and RoRγt (Fig. 2b and Additional file 4A). Most of these molecules were also significantly reduced in their splenic counterparts, except CTLA4 and GITR. FoxP3^+^ Tregs from *Prdm1*^fl/fl^*FoxP3*^YFP*-*Cre^ mice had more cells that were negative for both CD73 and FR4 (Fig. 2c). Since CD73^+^FR4^+^ cells are more anergic than CD73^−^FR4^−^ cells [23], the increased ratios of CD73^−^FR4^−^ Tregs to CD73^+^FR4^+^ Tregs from *Prdm1*^fl/fl^*FoxP3*^YFP*-*Cre^ mice suggested that Blimp1-deficient Tregs, particularly those in the spinal cords, displayed a more activated phenotype. Consistent with the increased expression of T_H_17 signature molecules (Fig. 2b), Blimp1-deficient Tregs expressed higher levels of IL-17A and GM-CSF, the critical encephalopathic T_H_17 effector cytokines [24, 25] (Fig. 2d, e). These results suggested that ablation of Blimp1 in Tregs destabilized and reprogrammed them to acquire T_H_17-like features in the face of neuroinflammation.

### Blimp1-deficient Tregs display impaired CNS Treg features in EAE mice and are insufficient to regulate CNS CD11b^+^ cells

**Fig. 3:**
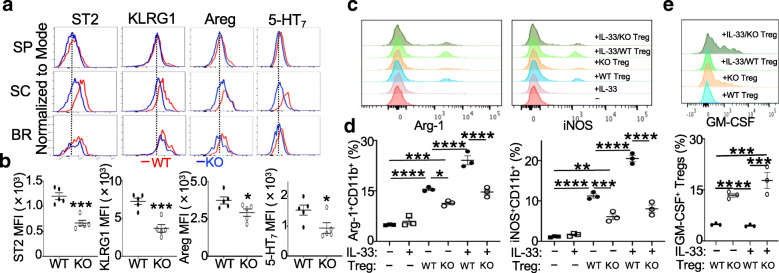
Blimp1-deficient Tregs display impaired CNS Treg features and are insufficient to regulate CNS CD11b^+^ cells. **a** Expression of indicated proteins in FoxP3^+^ Tregs from the spleen (SP), spinal cord (SC), and brain (BR) of mice at d20 post-EAE induction, as in Fig. 1a. **b** MFI of each protein expressed in SC Tregs in **a**. **c** Splenic YFP^+^(FoxP3^+^) Tregs and CNS CD45^+^CD11b^+^ cells were sorted from MOG-immune mice and were co-cultured in triplicates with or without IL-33 for 40 h. Histogram overlays of Arg-1 and iNOS in CD11b^+^ cells. **d** Frequencies of Arg-1^+^ or iNOS^+^ CD11b^+^ cells in **c**. **e** Histogram overlays of GM-CSF in Tregs in C. *Bottom*, frequencies of GM-CSF^+^ Tregs. WT, *FoxP3*^YFP-Cre^; KO, *Prdm1*^fl/fl^*FoxP3*^YFP-Cre^. Data represent one of two experiments (**b** *n* = 5/group). *P < 0.05, **P < 0.01, ***P < 0.001, and ****P < 0.0001 (**b**, unpaired two-tailed Student’s *t* test; **d**, **e**, one-way ANOVA post hoc Tukey’s multiple comparisons test). Bars, mean ± SEM

EAE recovery occurred in WT mice but not *Prdm1*^fl/fl^*FoxP3*^YFP*-*Cre^ mice (Fig. 1a). The impaired recovery was not a result of significantly fewer Blimp1-deficient Tregs in the CNS (Fig. 2a). We noted that compared to their splenic counterparts, Tregs from both brain and spinal cords expressed higher levels of several proteins related to tissue Tregs and resembled those CNS Tregs after ischemic stroke [19] (Fig. 3a). However, these proteins, ST2, KLRG1, Amphiregulin (Areg), and serotonin receptor (5-hydroxytryptamine receptor 7, 5-HT_7_), were expressed at lower levels in Blimp1-deficient Tregs than WT Tregs, particularly in the spinal cords, at day 20 post-EAE induction when WT mice underwent recovery from the disease (Fig. 3a, b and Additional file 4B). We also noted that the expression of tissue-resident markers, CD69 and CD103, was substantially reduced in CNS Tregs of *Prdm1*^fl/fl^*FoxP3*^Cre^ mice and this reduction was most obvious for the spinal cord Tregs (Additional file 4C). Interestingly, there were mild but statistically significant increases of both CD69 and CD103 in splenic Tregs of *Prdm1*^fl/fl^*FoxP3*^Cre^ mice compared to WT mice (Additional file 4C). These findings indicated that Blimp1 deficiency in Tregs altered their CNS-resident phenotype.

The CNS is highly enriched with IL-33-expressing cells and Tregs expressing the IL-33 receptor, ST2, regulate the tissue homeostasis [17, 19]. We noted that CD11b^+^ myeloid/microglia appeared to be more activated and less suppressive in *Prdm1*^fl/fl^*FoxP3*^YFP*-*Cre^ EAE mice (Fig. 1c–e), which led us to specifically evaluate the impact of the reduced expression of ST2 by Blimp1-deficient Tregs on their ability to shape the CNS-infiltrating and resident myeloid cells. CD45^+^CD11b^+^ myeloid cells (both microglia and infiltrating cells) were sorted from the brains and spinal cords of mice at day 10 after MOG immunization and then were co-cultured with Tregs sorted from spleens of these mice in the presence or absence of IL-33. This analysis revealed that CD11b^+^ myeloid cells had increased expression of Arg-1 and iNOS, two enzymes related to their suppressive activity, after co-cultured with both Tregs irrespective of their genotypes. However, their levels were significantly lower in the co-culture with Blimp1-deficient Tregs compared to WT Tregs (Fig. 3c, d). The addition of IL-33 in the co-culture with WT Tregs but not Blimp1-deficient Tregs increased the expression of both enzymes, which was independent of the direct role of IL-33 on myeloid cells (Fig. 3c, d), indicating that the increased expression of Arg-1 and iNOS by CD11b^+^ cells resulted from the effect of IL-33 on the Treg subsets expressing ST2. These results suggested that Blimp1-deficient Tregs were insufficient to induce the suppressive phenotype of CNS CD11b^+^ cells, at least partly due to the reduced IL-33/ST2 regulatory effects. We also noted that Blimp1-deficient Tregs in the co-culture expressed more GM-CSF than WT Tregs (Fig. 3e), indicating that deletion of Blimp1 in Tregs impaired their suppressive activity on CD11b^+^ cells, which was concomitant with their reprogramming into T_H_17 effector cells.

### Increased T_FH_-B-Ab response in *Prdm1*^fl/fl^*FoxP3*^YFP-Cre^ EAE mice

**Fig. 4:**
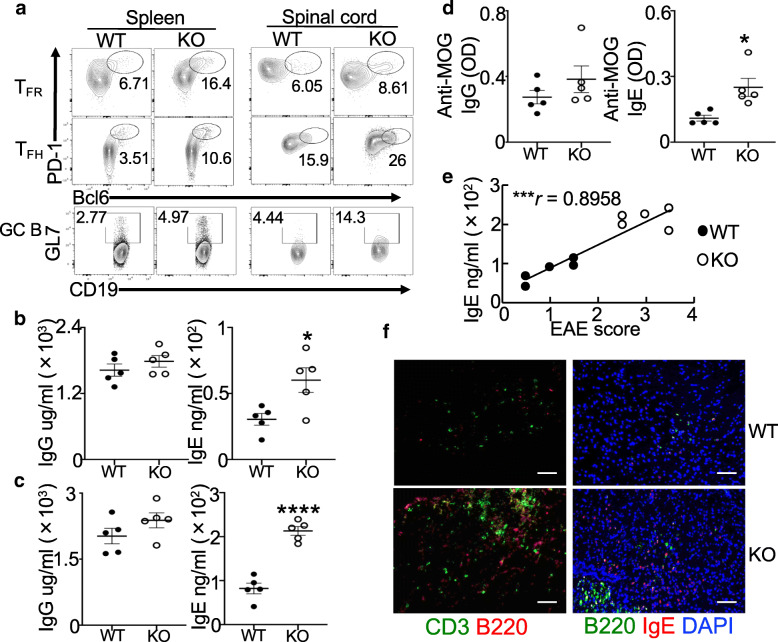
Increased T_FH_-B-Ab response in *Prdm1*^fl/fl^*FoxP3*^YFP-Cre^ EAE mice. **a** Flow cytometry of each indicated subset in mice at day 20 post-EAE induction, as in Fig. 1a. **b**–**d** Serum IgG and IgE at day 8 (**b**) or day 20 (**c**) or anti-MOG IgG and anti-MOG IgE at day 8 (**d**). **e** The relationship of serum IgE titers and EAE scores for mice in **c**. Each dot represents one mouse. **f** Immunofluorescence analysis of CD3, B220 (*left*) or B220, IgE and DAPI (*right*) in the spinal cords. Magnification, × 100. WT, *FoxP3*^YFP-Cre^, KO, *Prdm1*^fl/fl^*FoxP3*^YFP-Cre^. Data represent one of at least three experiments (**b**–**d** *n* = 5/group). *P < 0.05 and ****P < 0.0001 (unpaired two-tailed Student’s *t* test). In **e**, Spearman’s *r*, ***P = 0.0010. Bars, mean ± SEM

We next evaluated if ablation of Blimp1 in Tregs also affected T_FR_ cells and humoral Ab responses in MOG-EAE mice. We observed that T_FR_ cells (PD1^+^Bcl6^+^FoxP3^+^CD4^+^CD3^+^) were increased in both spleens and spinal cords of *Prdm1*^fl/fl^*FoxP3*^YFP*-*Cre^ mice compared to WT mice, albeit with no statistical significance in the spinal cords (Fig. 4a and Additional file 3A, B and 5A). T_FH_ cells (PD1^+^Bcl6^+^FoxP3^–^CD4^+^CD3^+^) and GL7^+^ B cells (GL7^+^CD19^+^) were also increased, which was more pronounced in the spinal cords of these mice (Fig. 4a and Additional file 5A). Consistent with the increased GC response, there were increased T_FH_ to T_FR_ ratios in both spleens and spinal cords, but the ratios only achieved statistical significance in the spinal cords of *Prdm1*^fl/fl^*FoxP3*^Cre^ mice compared to WT mice (Additional file 5B). We also observed that T_FR_, T_FH_, and non-T_FH_ cells (PD1^–^Bcl6^–^FoxP3^–^CD4^+^CD3^+^) in both spleens and spinal cords expressed low levels of IL-4, although T_FR_ cells had relatively higher proportion of cells expressing IL-4 than T_FH_ and non-T_FH_ cells (Additional file 6A, B). All of these cells from *Prdm1*^fl/fl^*FoxP3*^Cre^ mice had increased IL-4 expression compared to WT mice, especially for those from the spinal cords (Additional file 6A,B). The finding of increased IL-4 in Blimp1-deficient T_FR_ cells was consistent with our recent publication that also reports acquisition of T_FH_-like helper activity by unstable Blimp1-deficient T_FR_ cells [11]. Further analysis of T_FH_ phenotype revealed that T_FH_ cells from *Prdm1*^fl/fl^*FoxP3*^Cre^ EAE mice, particularly for those from the spinal cords, expressed higher levels of T_H_17 markers, including IL23R, CCR6, and RORγt (Additional file 6C, D). Consistently, these T_FH_ cells expressed more T_H_17 cytokines, IL-17A (except lower IL-17A in the spleens), and GM-CSF, but reduced levels of IFNγ (Additional file 6C-F). Interestingly, both splenic and spinal cord T_FH_ cells from *Prdm1*^fl/fl^*FoxP3*^Cre^ EAE mice expressed higher levels of intracellular CXCL13, a critical factor for the formation of GC and ectopic lymphoid structure (ELS) [26], than those from WT EAE mice (Additional file 6G).

We also noted that all of GC B cells in the spinal cords did not express substantial levels of CXCR4 compared to B cells in the spleens, and spinal cord GC B cells expressed higher levels of CD86, with a significantly higher expression in B cells from *Prdm1*^fl/fl^*FoxP3*^Cre^ mice (Additional file 6H), suggesting increased GC B cells with the dark zone phenotype in the spinal cords of these EAE mice. Consistent with the potentially increased proliferation and Ig gene somatic hypermutation by dark zone GC B cells [27], an increased intracellular IgE was observed in B cells of *Prdm1*^fl/fl^*FoxP3*^Cre^ EAE mice (Additional file 6I). These mice also had significantly higher levels of serum IgE and anti-MOG_35-55_ IgE (but not IgG or anti-MOG_35-55_ IgG) Abs than WT mice (starting at day 8 when *Prdm1*^fl/fl^*FoxP3*^YFP*-*Cre^ and WT mice had similar EAE severity) (Figs. 1a and 4b–d), and serum IgE titers were positively correlated with EAE scores (Fig. 4e). Although B cells rarely infiltrated the CNS of WT mice, there was a marked increase in B cells and T cells along with more IgE deposits in the spinal cords of *Prdm1*^fl/fl^*FoxP3*^YFP-Cre^ mice (Fig. 4f). Some IgE appeared to be expressed by B220^+^ cells, while others free from B220^+^ cells might be those IgE bound by its receptor on the surface of non-B cells (Fig. 4f). These findings suggested that deletion of Blimp1 in Tregs resulted in dysregulated T_FR_ and T_FH_ cells as well as the generation of abnormal Ab, particularly IgE and anti-MOG IgE.

### Increased activation of CNS CD11b^+^ cells after culture with serum from *Prdm1*^fl/fl^*FoxP3*^YFP-Cre^ EAE mice

**Fig. 5:**
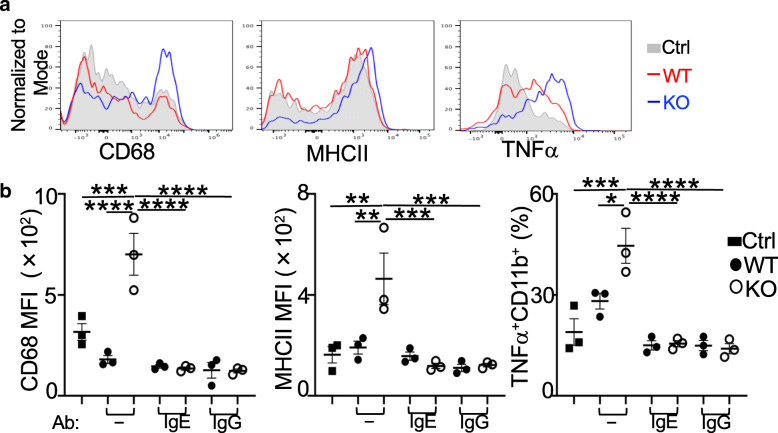
Increased activation of CNS CD11b^+^ cells after culture with serum from *Prdm1*^fl/fl^*FoxP3*^YFP-Cre^ EAE mice. **a** Sera from WT or KO EAE mice were added into the culture of CNS CD11b^+^ cells for 48 h. Flow cytometry of histogram overlays of CD68, MHCII, and TNFα in CD11b^+^ cells. **b** MFI of CD68 and MHCII, and frequencies of TNFα^+^ CD11b^+^ cells (as in **a**) cultured with or without sera in triplicates of each condition. Groups of cells pre-treated with anti-IgE or anti-IgG Ab were included. Ctrl, no mouse sera added; WT, sera from *FoxP3*^YFP-Cre^ EAE mice; KO, sera from *Prdm1*^fl/fl^*FoxP3*^YFP-Cre^ EAE mice. Data represent one of two experiments. *P < 0.05, **P < 0.01, ***P < 0.001, and ****P < 0.0001 (**b**, one-way ANOVA post hoc Tukey’s multiple comparisons test). Bars, mean ± SEM

The Ab deposition in the CNS may contribute to myeloid/microglia activation and EAE progression. The increased production of pro-inflammatory cytokines by microglia and less suppressive phenotype by CNS CD11b^+^ myeloid cells from *Prdm1*^fl/fl^*FoxP3*^YFP*-*Cre^ mice are suggestive of their activated status (Fig. 1c–e). We then explored if increased Ab production in these mice may promote myeloid/microglia activation using an in vitro culture assay. Myeloid/microglia isolated from adult WT mice were treated with a same volume of sera (5 μl, 2.5% of the total culture) collected from *Prdm1*^fl/fl^*FoxP3*^YFP*-*Cre^ or WT mice with ongoing EAE. It is technically challenging to isolate pure microglia from adult mice; about 15% CD11b^+^ myeloid/microglial cells from one mouse brain were obtained in our hands based on a published isolation method [21]. The addition of *Prdm1*^fl/fl^*FoxP3*^YFP*-*Cre^ EAE sera but not WT sera increased the expression of CD68 and MHCII on CD11b^+^ cells as well as the proportion of CD11b^+^ cells expressing TNFα (Fig. 5a, b), suggestive of an increased activation of CD11b^+^ cells. Notably, pre-incubation of sera with either anti-IgG or anti-IgE to neutralize their activity prior to the culture largely diminished the upregulation of MHCII, CD68, and TNFα in CD11b^+^ cells (Fig. 5a, b). These findings suggested that sera from *Prdm1*^fl/fl^*FoxP3*^YFP*-*Cre^ EAE mice, at least partly due to more IgG or IgE Ab included (albeit with other factors non-excluded), had the potential to increase the activation and production of pro-inflammatory cytokines by CNS CD11b^+^ cells.

### Transfer of Blimp1-deficient T_FR_ promotes EAE

**Fig. 6:**
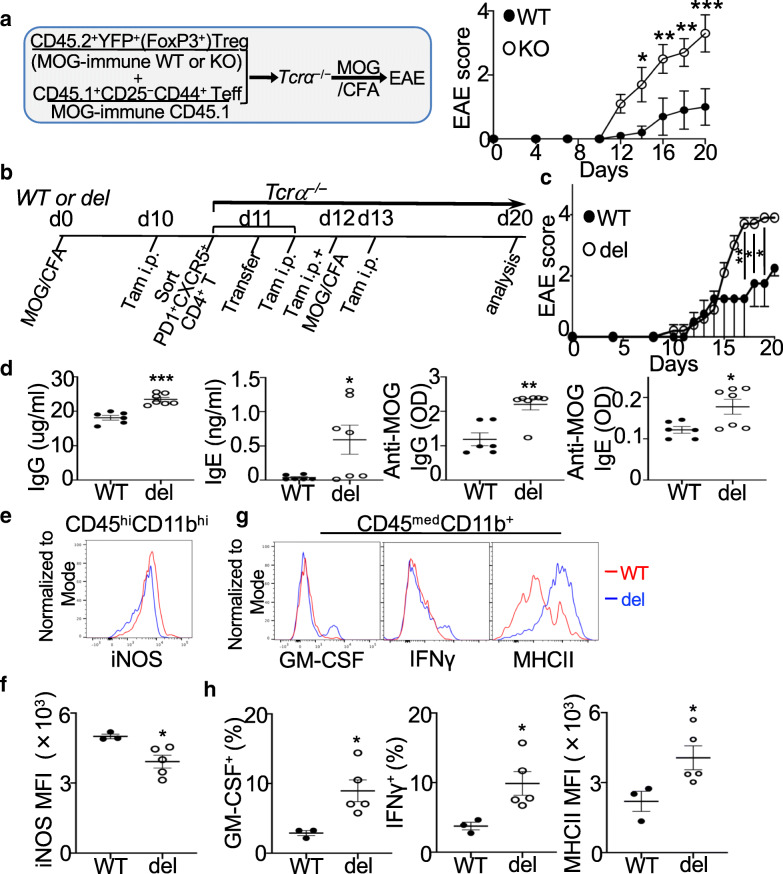
Transfer of Blimp1-deficient T_FR_ cells promotes EAE. **a** Schematic presentation of adoptive transfer assay (*left*) and EAE scores in recipient mice (*right*). WT, *FoxP3*^YFP-Cre^; KO, *Prdm1*^fl/fl^*FoxP3*^YFP-Cre^. **b**–**h** Schematic diagram of experiment (**b**), EAE scores in recipient mice (**c**); serum IgG and IgE or anti-MOG IgG and anti-MOG IgE at day 20 (**d**), iNOS expression in brain CD45^hi^CD11b^hi^ cells (**e**, **f**), and GM-CSF, IFNγ, and MHCII expression in brain microglia (CD45^med^CD11b^+^) of recipients (**g**, **h**)**.** WT, *Rosa26*^ERT2-Cre^; del, *Prdm1*^fl/fl^*Rosa26*^ERT2-Cre^. Data represent one of two experiments (**a** *n* = 5/group; **c**, **f**, **h** *n* = 3–5/group). **d** Data are pooled from two independent experiments. *P < 0.05, **P < 0.01, and ***P < 0.001 (**a**, **c**, two-way ANOVA post hoc Sidak’s multiple comparisons test; **d**, **f**, **h**, unpaired two-tailed Student’s *t* test). Bars, mean ± SEM

We next asked whether Blimp1-deficient Tregs contributed to EAE in a cell-intrinsic manner using the adoptive transfer approach. CD45.2^+^ Tregs sorted from MOG-immune *Prdm1*^fl/fl^*FoxP3*^YFP*-*Cre^ mice or WT mice along with CD45.1^+^CD25^−^CD44^+^CD4^+^Teff were transferred into *Tcrα*^−/−^ hosts followed by EAE induction. Mice transferred with Blimp1-deficient Tregs induced more severe disease compared to mice given WT Tregs (Fig. 6a), suggesting that Blimp1-deficient Tregs facilitated EAE.

Blimp1^+^ Tregs comprise both T_FR_ cells and conventional non-T_FR_ Tregs. Our recent publication has indicated that Blimp1-deficient non-T_FR_ Tregs do not contribute significantly to the increased frequency of T_FH_ and GCB cells or dysregulated Ab responses observed in *Prdm1*^fl/fl^*FoxP3*^Cre^ mice [11]. Instead, Blimp1-deficient T_FR_ cells are capable of supporting GC-Ab response due to the acquisition of T_FH_-like properties post-immunization [11]. To finally define the contribution of Blimp1^+^ T_FR_ cells independent of other Tregs to the regulation of Ab responses and EAE, we used an inducible Blimp1 deletion system to circumvent potential developmental defects secondary to inflammation or other changes in the environment. We generated *Prdm1*^fl/fl^*Rosa26*^Cre-ERT2^ (del) or *Rosa26*^Cre-ERT2^ (WT) control mice to allow deletion of Blimp1 after administration of tamoxifen. PD1^+^CXCR5^+^CD4^+^ T cells (both Blimp1^+^ T_FR_ and Blimp1^−^ T_FH_) were sorted from *Prdm1*^fl/fl^*Rosa26*^Cre-ERT2^ mice or *Rosa26*^Cre-ERT2^ (WT) mice 10 days after MOG immunization and 1 day after tamoxifen administration. These cells were then transferred into *Tcra*^–/–^ mice before EAE induction and injection of tamoxifen for 3 more days (Fig. 6b). This method can substantially reduce Blimp1 expression specifically by T_FR_ cells from *Prdm1*^fl/fl^*Rosa26*^Cre-ERT2^ mice along with increased T_FR_, T_FH_, and GC B cells [11]. Although transfer of in vitro differentiated T_FH_ cells alone fails to induce EAE [4], our transfer system included both ex vivo isolated T_FH_ and T_FR_ cells that were able to induce EAE (Fig. 6c). We observed that *Tcrα*^−/−^ mice transferred with Blimp1-deleted T_FR_ cells had an increased disease severity associated with increased total and MOG-specific IgG and IgE compared to mice transferred with WT T_FR_ cells (Fig. 6c, d). Interestingly, CNS-infiltrating CD45^hi^CD11b^hi^ cells expressed significantly lower levels of iNOS (Fig. 6e, f), while CD45^med^CD11b^+^ microglia expressed higher levels of MHCII and more GM-CSF^+^ and IFNγ^+^ microglia were observed in mice given Blimp1-deleted T_FR_ cells than mice transferred with WT T_FR_ cells (Fig. 6g, h), consistent with our analysis of *Prdm1*^fl/fl^*FoxP3*^Cre^ EAE mice and in vitro culture assays (Figs. 1c–e, 3c, d, and 5). Taken together, these results suggested that Blimp1-deleted T_FR_ cells were more encephalitogenic than WT T_FR_ cells, contributing to dysregulated Ab responses, hyperactivation of CNS CD11b^+^ cells, and subsequently EAE progression.

## Discussion

T_FR_ cells represent a phenotypically and functionally specialized Treg population that controls the cellular and humoral immune response. Due to the low frequency of this Treg population, its role in the regulation of immune response has been underappreciated until the recent findings using T_FR_-deleter mice [10]. Our recent publication has further confirmed the importance of T_FR_ cells in the maintenance of humoral self-tolerance [11]. Results obtained from this study reveal that dysregulated T_FR_ responses contribute to CNS autoimmune diseases. The heightened EAE responses reflect the reprogramming of Tregs into Teff with enhanced T_H_17 activity along with the abnormal expansion of T_FH_/B cells and increased Ab production secondary to impaired T_FR_ suppression.

Tregs, including T_FR_ cells, must maintain their suppressive anergic phenotype during ongoing inflammatory responses [11, 13, 28]. This functional stability reflects a lack of effector activity by Tregs (i.e., expression of pro-inflammatory cytokines) and may or may not require stable FoxP3 expression. Loss of FoxP3 (even slight reductions) often results in generation of exTregs [29, 30], while conversion into Teff with unaltered FoxP3 expression is called Treg “fragility” [31]. Several factors appear to be important for Treg stability/fragility, including IL-2/STAT5 signals [11, 28, 32], Pten/Akt/Foxo1/3a pathway [33, 34], autophagy [35], CARMA1–BCL10–MALT1 (CBM) signalosome complex [36], Ezh2 [37], Bcl11b [38], Eos [39], PP2A [40], and Nrp1 [31]. While the former six pathways are required to stabilize FoxP3, ablation of the latter three factors does not affect FoxP3 expression. Our recent publication has revealed that the expression of Blimp1 by Tregs is essential for the maintenance of FoxP3 expression and effector Treg (but not central Treg) lineage stability, in part through regulation of the IL-2/STAT5 pathway [11]. Consistently, here we observed that Blimp1-deficient Tregs converted into T_H_17-like CD4^+^ Teff in EAE mice and transfer of Blimp1-deficient Tregs or T_FR_ cells exacerbated EAE. Although Blimp1 expression in Tregs has been recently reported to regulate EAE or other autoimmune disorders [41–43], our study is the first to reveal the potential role of Blimp1^+^T_FR_ cells in the regulation of EAE. Moreover, the finding that Blimp1-deficient T_FR_ cells displayed T_H_17 phenotype and expressed elevated levels of T_H_17 cytokines may provide partial explanations for the reported increased T_H_17-like phenotype with reduced suppressive function by circulating T_FR_ cells in MS patients compared to HC [14]. T_FR_ cells with reduced Blimp1 expression may provide additional sources of heightened T_H_17 activity to promote EAE and MS. Although we cannot exclude the possibility that other factors may cause fewer circulating T_FR_ cells in MS patients rather than increased splenic T_FR_ cells as observed in mice with a Treg-specific deletion of Blimp1, our study has uncovered Blimp1 as a new regulator that is important for Treg stability during CNS autoimmunity, and has established the in vivo pathological importance of Treg conversion into T_H_17-like cells.

The mechanisms for the Blimp1-dependent regulation of stable Treg response to CNS autoimmunity are likely disease stage- and tissue-specific. Blimp1 does not directly bind to the *FoxP3* loci [11, 41], and Blimp1 expression in Tregs has been recently shown to prevent methylation of *FoxP3* by counteracting IL-6-driven loss of FoxP3 at the peak of EAE [41]. Because stable FoxP3 expression in Tregs may resume with the resolution of inflammation [44], it is unclear if the above mechanism remains operational during the remission phase of the disease. Despite that complete demethylation of the *conserved non-coding sequence 2* (*CNS2*), also known as Treg cell-specific demethylated region (TSDR), in the first intron of the *FoxP3* locus is required for optimal expression of FoxP3 [13, 45], Tregs with a fully demethylated TSDR still lose FoxP3 expression and become exTregs in a MOG-EAE model [44], suggesting that lack of epigenetic imprinting in the *CNS2* may not always be the leading cause of FoxP3 loss in Tregs during EAE [44]. The above considerations along with our finding that *Prdm1*^fl/fl^*FoxP3*^YFP-Cre^ mice failed to recover from EAE suggest that stage-specific genetic and epigenetic elements may regulate Blimp1-dependent Treg stability during EAE. Moreover, both peripheral and CNS Blimp1-deficient Tregs adopted a T_H_17-like phenotype, but the unstable phenotype indicative of loss of anergy was more pronounced in the CNS. This finding suggests that CNS Treg reprogramming may reflect the collaborative effects of Blimp1 deficiency and CNS-specific factors, and future studies are warranted to define such potential factors. The downregulation of several CNS Treg markers by Blimp1-deficient Tregs and their reduced ability to respond to IL-33 in the regulation of CNS myeloid cells also support that Blimp1 may be required to establish the CNS Treg signature and to maintain CNS homeostasis in the face of neuroinflammation. The upregulation of CD69 and CD103 in splenic Tregs but downregulation in CNS Tregs may result in the retention of Tregs in the periphery, consistent with the increased splenic Tregs noted in *Prdm1*^fl/fl^*FoxP3*^Cre^ EAE mice compared to WT mice. However, frequencies of Tregs were only slightly reduced in the spinal cords of *Prdm1*^fl/fl^*FoxP3*^Cre^ EAE mice, indicating that detailed analyses are needed in order to precisely determine the distribution of these Tregs in the context of EAE.

Other mechanisms may exist for the impaired stability and suppressive activity of Blimp1-deficient Tregs. Blimp1 is essential for the production of IL-10, and reduced IL-10 may contribute to the impaired suppressive activity of Blimp1-deficient Tregs. However, we and others have shown that Blimp1-dependent regulation of Treg stability is IL-10-independent [11, 41]. The increased IL-17A production by Blimp1-deficient Tregs is likely secondary to the reduced FoxP3 expression, but may also result from the lack of Blimp1-mediated inhibition of IL-17 expression due to the absent occupancy of Blimp1 on the *Il17* locus [43]. Blimp1 may also regulate other factors that are implicated in Treg suppression. We noted reduced expression of CD73 on Blimp1-deficient Tregs that may result in reduced adenosine production, contributing to the overall impaired immunosuppression. Additionally, we have shown that increased IL23R expression and STAT3 activation contribute to Blimp1-deficient Treg instability [11]. These Tregs are likely sensitive to IL-23 expressed in the inflamed CNS to promote EAE [46, 47], which may suggest a molecular link between Blimp1 and EAE/MS and support the association of *IL23R* and *STAT3* gene variants with increased risk for MS [48, 49]. Moreover, the reduced expression of ST2 and decreased response to IL-33 by Blimp1-deficient Tregs may further enhance their encephalitogenicity. Future studies are required to define which signals are altered in Tregs and T_FR_ cells that lead to reduced Blimp1 expression, impaired stability, and suppressive activity in the context of EAE.

Clonally related B cells and plasma cells are commonly found in active MS plaques [6]. However, the functional effects of B cells on EAE and MS have been inconsistently reported, largely due to B cell heterogeneity and inappropriate experimental approaches used in some studies. For example, the CD20 receptor does not mark plasma cells and there are many different B cell subsets with either pro-inflammatory or regulatory activity. The CD20-mediated B cell depletion may also reduce the frequency of Tregs associated with the enhanced pro-inflammatory function of myeloid antigen-presenting cells (APCs) [50]. The use of μMT mice that lack B cells is also inappropriate since these mice have severe immune abnormalities [51]. Our analysis of B cells in EAE mice further supports that dysregulated B cells may promote EAE. Here we mainly focus on the Ab-dependent role of B cells, but we cannot exclude the possibility that B cells may regulate autoimmunity via operating as APCs to enhance T cell response or secreting GM-CSF [52]. Our experiments also cannot distinguish if regulation of B-Ab responses by T_FR_ cells is directly or indirectly mediated via suppression of T_FH_ cells.

Although the presence of cerebrospinal fluid (CSF)-specific antibodies, mainly IgG, is a hallmark in the diagnosis of MS [6, 7], the nature of these antibodies remains unclear. Our finding that mice with a deletion of Blimp1 in Tregs display high titers of IgE, including anti-MOG IgE, and increased IgE deposition in the inflamed CNS is unexpected. However, given that T_H_17 cells are effective B cell helpers and IL-17^+^ cells promote IgE production by acting on B cells [15, 53], the increased production of IL-17A by both T_FH_ and T_FR_ cells in *Prdm1*^fl/fl^*FoxP3*^Cre^ EAE mice may enable them to regulate B cells and help IgE production, which may be enhanced by low levels of IL-4 expression from both cell types in these EAE mice. Although further study is required to confirm these propositions, the dysfunctional T_FH_ and T_FR_ cell phenotype suggests that abnormal GC responses with increased IgE production in *Prdm1*^fl/fl^*FoxP3*^Cre^ EAE mice may result from both dysregulated T_FH_ and T_FR_ cells that had elevated B cell helper activity due to increased IL-17A and IL-4 expression. Moreover, in light of a recent finding that T_FR_ cell-deleter mice develop high levels of autoreactive IgE [10], our study suggests that induction of T_FR_ instability, in addition to decreasing its numbers, is capable of boosting B cell/IgE Ab response. The genetic and functional status of T_FR_ cells, in addition to their numbers, should all be considered when evaluating GC responses.

Although IgE titers are lower than IgG in EAE mice, IgE may synergize the effects of IgG or induce specific effects, e.g., phagocytosis, to augment autoimmunity [54]. The production of anti-MOG antibodies is thought not to take place in the MOG_35–55_-induced EAE model in a meaningful manner. However, the potentially pathogenic role of autoantibodies in this model may have been overlooked [5]. Given the increased total IgE in *Prdm1*^fl/fl^*FoxP3*^Cre^ mice and adoptive hosts with EAE, IgE autoantibodies other than anti-MOG IgE may also contribute to EAE. Considering the recent finding that IgE autoantibodies contribute to autoimmunity without allergic manifestation [54–56], the ongoing therapy for other autoimmune disorders using IgE blocker (omalizumab) [54, 57], and the positive correlation of serum IgE titers with EAE scores reported in this study as well as increased IgE in some MS patients from other reports [58, 59], understanding of its direct contribution to EAE and MS is of key importance. Thus, as a proof-of-concept, mice with high levels of serum IgE secondary to the Treg-specific deletion of Blimp1 are useful models for further characterizing the autoreactivity and encephalitogenicity of IgE and defining the regulatory mechanisms for IgE production. Future comprehensive and longitudinal analysis of MS specimens with different disease severity, including analysis of IgE levels and specificity in the sera and CSF of MS patients, may also help suggest diagnostic or prognostic markers for MS.

The increased T and B cells in the CNS of *Prdm1*^fl/fl^*FoxP3*^YFP-Cre^ mice may suggest the potential formation of ELS that is well established in MS autopsy and biopsy [60]. Currently, it remains unclear about the cellular composition of ELS that is likely disease stage-specific and experimental model-dependent. It may only comprise disordered mixtures of dendritic cell (DC)/B/T cells [60], but not a well-organized GC-like structure. The presence of Tregs in the ELS may also vary, and the ELS in patients with progressive MS does not have Tregs in the brain [61]. The converted Blimp1-deficient Tregs that express T_H_17 cytokines may promote ELS formation [4, 15, 62]. Indeed, the increased expression of CXCL13 in T_FH_ cells and the increased GC B cells with the dark zone phenotype as well as more B cells expressing intracellular IgE in the spinal cords of *Prdm1*^fl/fl^*FoxP3*^YFP-Cre^ EAE mice suggest the presence of reactive GC-like responses. However, the cellular composition and distribution of ELS in the CNS of these mice require further analysis.

Blimp1-deficient Tregs produced more GM-CSF and IL-17A (but not IFNγ) than WT Tregs. FoxP3^−^CD4^+^Teff cells from *Prdm1*^fl/fl^*FoxP3*^YFP-Cre^ mice, including T_FH_ cells, also expressed more GM-CSF. No differences in GM-CSF and IL-17A expression in CD8^+^ T cells and NK cells were noted, and IFNγ was reduced in these effector cells in *Prdm1*^fl/fl^*FoxP3*^YFP-Cre^ mice. Although expression of IL-17 or its cognate receptor is dispensable for the manifestation of active EAE [63], and disruption of GM-CSF signaling in adoptive transfer recipients does not reduce the incidence or mitigate the early clinical course of EAE [64], it remains unclear if IL-17A produced from Blimp1-deficient Tregs facilitates EAE, or if GM-CSF produced from Blimp1-deficient Tregs augments disease severity/chronicity and prevents remission. Interestingly, microglia from *Prdm1*^fl/fl^*FoxP3*^YFP*-*Cre^ mice and mice given Blimp1-deleted T_FR_ cells expressed higher levels of GM-CSF than WT mice. Serum from *Prdm1*^fl/fl^*FoxP3*^YFP*-*Cre^ EAE mice also enhanced TNFα production by myeloid/microglial cells, indicative of activation. Activated microglia are known to promote EAE and MS [65]. Although GM-CSF signaling in microglia is not critical for EAE development [66], further analysis is warranted to define if GM-CSF produced by microglia contribute to EAE by regulating Tregs or other immune cells and if microglia from *Prdm1*^fl/fl^*FoxP3*^YFP*-*Cre^ mice display a “neurotoxic” profile [67] that regulates demyelination or remyelination process during CNS autoimmunity.

## Conclusions

In summary, this study has not only explored the importance of Blimp1 in maintaining Treg/T_FR_ cell lineage in the context of EAE but also addressed how CNS homeostasis and EAE disease activity are modulated by T_FR_ and GC Ab response. Treg-based therapies are being tested in clinical trials in other autoimmune disorders, where Treg instability that leads to unwarranted effects in patients is one of the major concerns. The presence of T_H_17-like exTregs may be used as a biomarker for MS, contributing to future diagnostic and therapeutic strategies for MS. Moreover, the finding that Blimp1 loss in Tregs and T_FR_ cells promotes CNS autoimmunity may suggest new approaches to manipulation of Treg activity in vivo and provide critical strategies to formulate new or combined therapeutic approaches to MS and other autoimmune disorders.

## Supplementary Information

**Additional file 1.** Table 1: Antibodies used for flow cytometry analysis.

**Additional file 2 **Table 2: EAE disease parameters. Data are pooled from there independent experiments and are presented as mean ± SD. **P < 0.01 and ****P < 0.0001 ( unpaired two-tailed Student’s t-test). -, not available.

**Additional file 3 **Gating strategy for analysis of splenic CD4/B cells (**A**), spinal cord CD4/B cells (**B**) and brain myeloid/microglial cells (**C**).

**Additional file 4 **Blimp1-deficient Tregs are unstable and display impaired CNS Treg features in EAE mice. **A**) MFI of each molecule in SP and SC FoxP3^+^ Tregs from mice at day 20 post-EAE induction in Fig. 2b. **B**) MFI of each protein expressed in spleen (SP) and brain (BR) Tregs, as in Fig. 3a. **C-D**) Expression of CD103 (**C**) and CD69 (**D**) in FoxP3^+^ Tregs from the spleen (SP), spinal cord (SC) and brain (BR) of mice at d20 post-EAE induction, as in Fig. 1a. *Right*, MFI of each protein. WT: *FoxP3*^YFP-Cre^; KO: *Prdm1*^fl/fl^*FoxP3*^YFP-Cre^. *P < 0.05, **P < 0.01, ***P < 0.001 and ****P < 0.0001 (unpaired two-tailed Student’s t-test). Bars, mean ± SEM.

**Additional file 5 **T_FR_, T_FH_ and B cells in EAE mice. **A**) Frequencies of T_FR_, T_FH_ and GL7^+^ B cells in spleen (SP) and spinal cord (SC) of mice, as in Fig. 4a. **B**) Ratios of T_FH_ : T_FR_ of CD4^+^ T cells from SP and SC of each mouse in Fig. 4a. WT: *FoxP3*^YFP-Cre^, KO: *Prdm1*^fl/fl^*FoxP3*^YFP-Cre^. *P < 0.05 (unpaired two-tailed Student’s t-test). Bars, mean ± SEM.

**Additional file 6 **Analysis of T_FH_ and B cells in the spleens and spinal cords of EAE mice. **A-B**) Flow cytometry (**A**) and frequencies (**B**) of intracellular IL-4 expression in non-T_FH_ (PD-1^−^Bcl6^−^FoxP3^−^CD4^+^CD3^+^), T_FH_ (PD-1^+^Bcl6^+^FoxP3^−^CD4^+^CD3^+^) and T_FR_ cells (PD-1^+^Bcl6^+^FoxP3^+^CD4^+^CD3^+^) from spleen (SP) and spinal cord (SC) of EAE mice, as in Fig. 1a. **C-D**) Expression (**C**) and quantitation of MFI of each molecule in T_FH_ cells or frequencies of IFNγ^+^ T_FH_ cells (**D**) from mice in A. **E-F**) Frequencies of IL-17A^+^ (**E**) and GM-CSF^+^ (**F**) T_FH_ cells from mice in A. **G**) Histogram overlays of intracellular CXCL13 in T_FH_ or splenic non-T_FH_ cells from mice in A. *Right*, MFI of CXCL13. **H**) Histogram overlays of CD86 or CXCR4 in GC B-cells (GL-7^+^Fas^+^IgD^−^CD19^+^) from mice in A. *Right*, MFI of CD86. **I**) Flow cytometry of intracellular IgE expression in IgD^−^CD19^+^ B cells (*left*) and frequencies of IgE^+^IgD^−^CD19^+^ B-cells (*right*) from mice in A. WT: *FoxP3*^YFP-Cre^; KO: *Prdm1*^fl/fl^*FoxP3*^YFP-Cre^. In A-I, *n* = 4/group, except *n* = 4-5/group in D. *P < 0.05, **P < 0.01 and ***P < 0.001 (unpaired two-tailed Student’s t-test). Bars, mean ± SEM.

## Data Availability

All data generated or analyzed during this study are included in this article [and its supplementary information (Additional files)].

## Abbreviations

Ab: Antibody
APC: Antigen-presenting cells
Areg: Amphiregulin
Arg-1: Arginase-1
CFA: Complete Freund’s adjuvant
CNS: Central nervous system
CNS2: Conserved non-coding sequence 2
CSF: Cerebrospinal fluid
DC: Dendritic cell
DMEM: Dulbecco’s modified Eagle medium
EAE: Experimental autoimmune encephalomyelitis
ELISA: Enzyme-linked immunosorbent assay
ELS: Ectopic lymphoid structure
FACS: Flow-activated cell sorting
FBS: Fetal bovine serum
GC: Germinal center
GM-CSF: Granulocyte-macrophage colony-stimulating factor
HC: Healthy controls
5-HT_7_: 5-Hydroxytryptamine receptor 7
IL: Interleukin
iNOS: Inducible nitric oxide synthase
MFI: Mean fluorescence intensity
MHCII: Major histocompatibility complex class II
MOG: Myelin oligodendrocyte glycoprotein
MS: Multiple sclerosis
PBS: Phosphate-buffered saline
SD: Standard deviation
SEM: Standard error of the mean
TCR: T cell receptor
Teff: Effector T cells
TF: Transcription factor
T_FH_: Follicular helper T cells
T_FR_: Follicular regulatory T cells
Treg: Regulatory T cell
TSDR: Treg cell-specific demethylated region
