# Supplementary figures and images for: Dysregulated follicular regulatory T cells and antibody responses exacerbate experimental autoimmune encephalomyelitis
Source: J Neuroinflammation. 2021 Jan 19;18:27. doi: 10.1186/s12974-021-02076-4 (PMC7814531; doi:10.1186/s12974-021-02076-4)

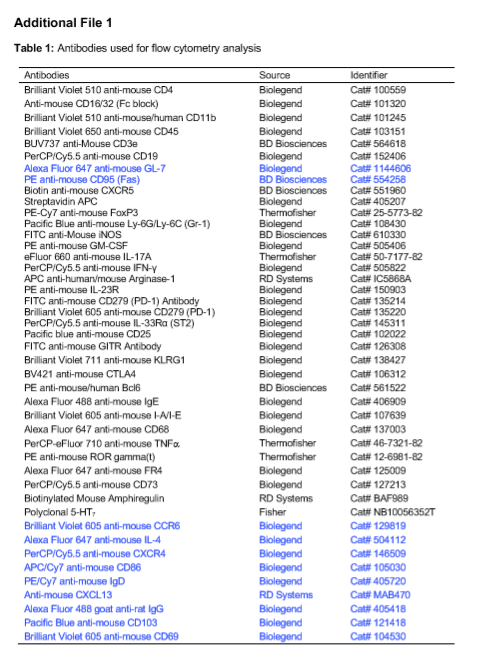

Supplement: Supplementary file 1 — Additional file 1. Table 1: Antibodies used for flow cytometry analysis. [file 12974_2021_2076_MOESM1_ESM.docx]

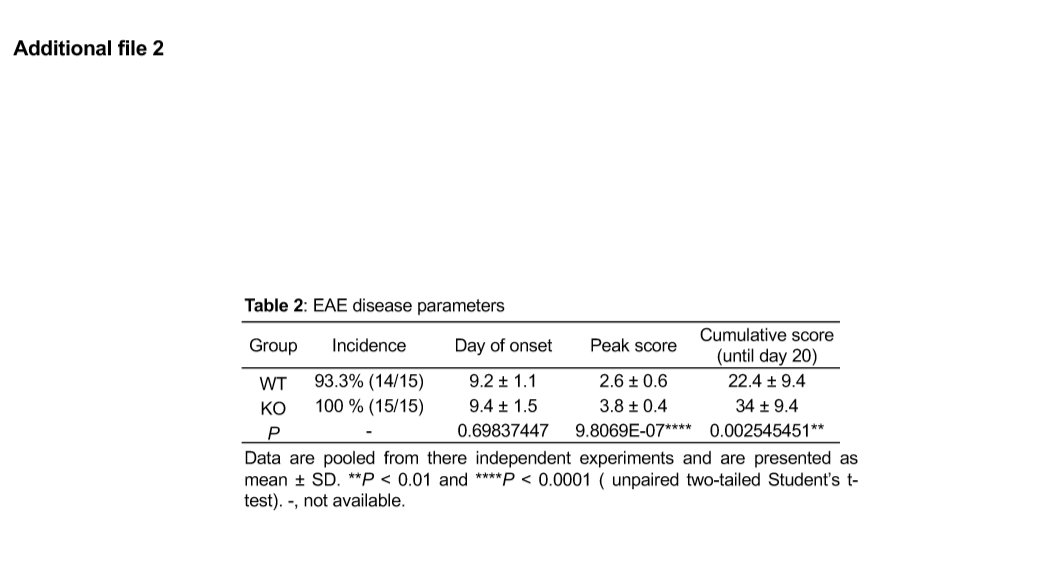

Supplement: Supplementary file 2 — Additional file 2 Table 2: EAE disease parameters. Data are pooled from there independent experiments and are presented as mean ± SD. **P < 0.01 and ****P < 0.0001 ( unpaired two-tailed Student’s t-test). -, not available. [file 12974_2021_2076_MOESM2_ESM.docx]

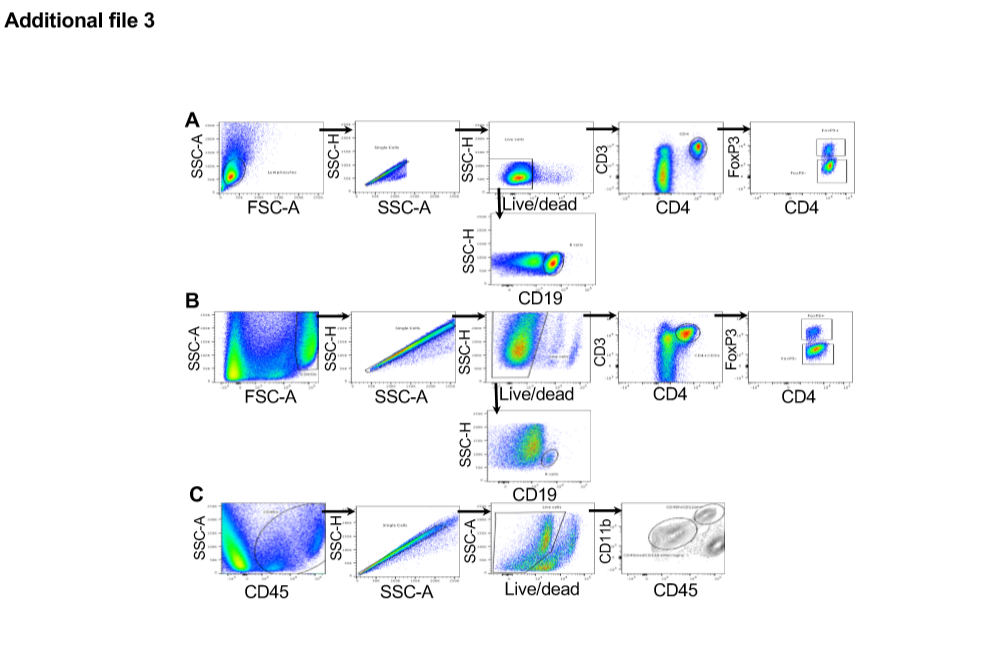

Supplement: Supplementary file 3 — Additional file 3 Gating strategy for analysis of splenic CD4/B cells (A), spinal cord CD4/B cells (B) and brain myeloid/microglial cells (C). [file 12974_2021_2076_MOESM3_ESM.docx]

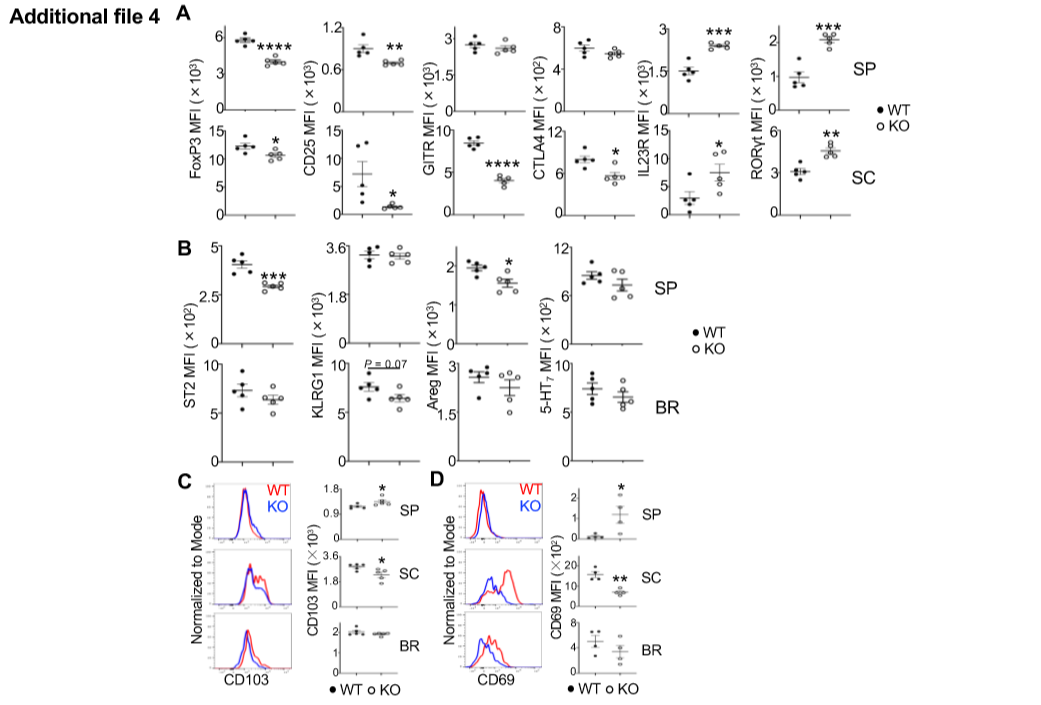

Supplement: Supplementary file 4 — Additional file 4 Blimp1-deficient Tregs are unstable and display impaired CNS Treg features in EAE mice. A) MFI of each molecule in SP and SC FoxP3+ Tregs from mice at day 20 post-EAE induction in Fig. 2b. B) MFI of each protein expressed in spleen (SP) and brain (BR) Tregs, as in Fig. 3a. C-D) Expression of CD103 (C) and CD69 (D) in FoxP3+ Tregs from the spleen (SP), spinal cord (SC) and brain (BR) of mice at d20 post-EAE induction, as in Fig. 1a. Right, MFI of each protein. WT: FoxP3YFP-Cre; KO: Prdm1fl/flFoxP3YFP-Cre. *P < 0.05, **P < 0.01, ***P < 0.001 and ****P < 0.0001 (unpaired two-tailed Student’s t-test). Bars, mean ± SEM. [file 12974_2021_2076_MOESM4_ESM.docx]

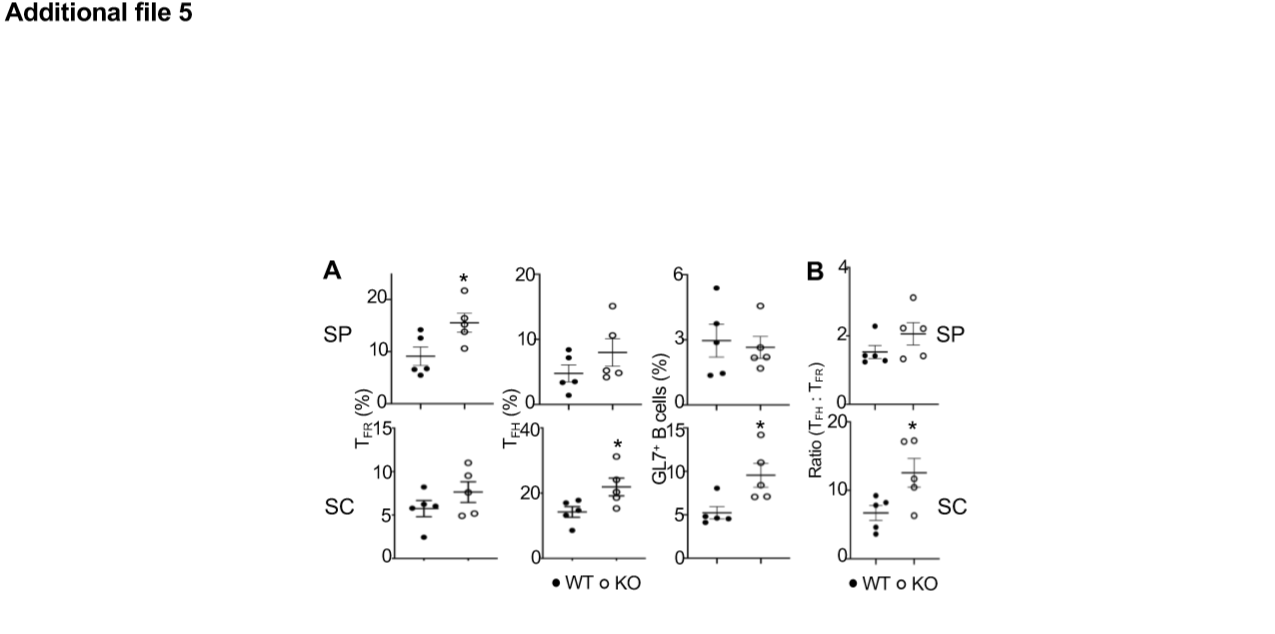

Supplement: Supplementary file 5 — Additional file 5 TFR, TFH and B cells in EAE mice. A) Frequencies of TFR, TFH and GL7+ B cells in spleen (SP) and spinal cord (SC) of mice, as in Fig. 4a. B) Ratios of TFH : TFR of CD4+ T cells from SP and SC of each mouse in Fig. 4a. WT: FoxP3YFP-Cre, KO: Prdm1fl/flFoxP3YFP-Cre. *P < 0.05 (unpaired two-tailed Student’s t-test). Bars, mean ± SEM. [file 12974_2021_2076_MOESM5_ESM.docx]

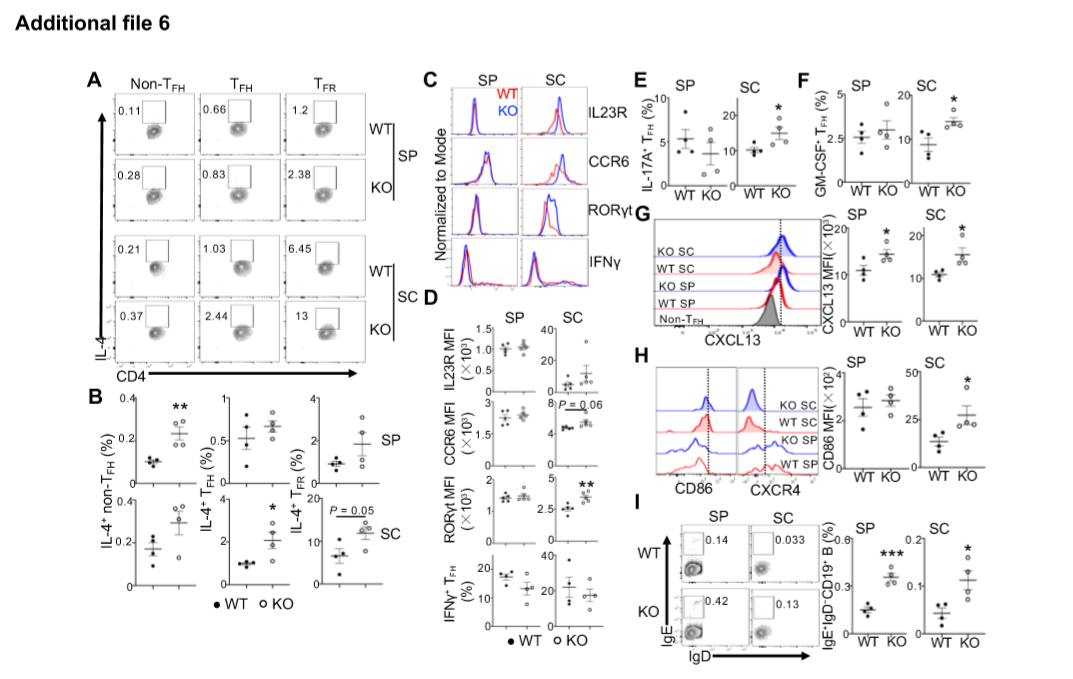

Supplement: Supplementary file 6 — Additional file 6 Analysis of TFH and B cells in the spleens and spinal cords of EAE mice. A-B) Flow cytometry (A) and frequencies (B) of intracellular IL-4 expression in non-TFH (PD-1−Bcl6−FoxP3−CD4+CD3+), TFH (PD-1+Bcl6+FoxP3−CD4+CD3+) and TFR cells (PD-1+Bcl6+FoxP3+CD4+CD3+) from spleen (SP) and spinal cord (SC) of EAE mice, as in Fig. 1a. C-D) Expression (C) and quantitation of MFI of each molecule in TFH cells or frequencies of IFNγ+ TFH cells (D) from mice in A. E-F) Frequencies of IL-17A+ (E) and GM-CSF+ (F) TFH cells from mice in A. G) Histogram overlays of intracellular CXCL13 in TFH or splenic non-TFH cells from mice in A. Right, MFI of CXCL13. H) Histogram overlays of CD86 or CXCR4 in GC B-cells (GL-7+Fas+IgD−CD19+) from mice in A. Right, MFI of CD86. I) Flow cytometry of intracellular IgE expression in IgD−CD19+ B cells (left) and frequencies of IgE+IgD−CD19+ B-cells (right) from mice in A. WT: FoxP3YFP-Cre; KO: Prdm1fl/flFoxP3YFP-Cre. In A-I, n = 4/group, except n = 4-5/group in D. *P < 0.05, **P < 0.01 and ***P < 0.001 (unpaired two-tailed Student’s t-test). Bars, mean ± SEM. [file 12974_2021_2076_MOESM6_ESM.docx]
